# Supplementary material for: Morphological and Phylogenetic Analyses Reveal Three New Species of Entomopathogenic Fungi Belonging to Clavicipitaceae (Hypocreales, Ascomycota)
Source: J Fungi (Basel). 2024 Jun 14;10(6):423. doi: 10.3390/jof10060423 (PMC11204714; doi:10.3390/jof10060423)
Supplement: Supplementary file 1 [file jof-10-00423-s001.zip › jof-2864033-supplementary.pdf]

Table S1. Interspecific genetic distance matrix and intragroup genetic distances for nr*LSU* sequences of *Moelleriella*.

Species	1	2	3	4	5	6	7	8	9	10	11	12	13	14	15	16	17	18	19	20	21	22	23	24	25	26	27	28	29	30	31	32	33	34	Genetic distance (within groups)
1.*Moelleriella phyllogena*																																			0.0026
2.*M. pongdueatensis*	0.04																																		0
3.*M. raciborskii*	0.04	0.04																																	0.0024
4.*M. rhombispora*	0.04	0.05	0.04																																0.0012
5.*M. schizostachyi*	0.06	0.06	0.06	0.06																															n/c
6.*M. sloaneae*	0.04	0.04	0.04	0.04	0.05																														0
7.*M. turbinata*	0.08	0.07	0.07	0.07	0.08	0.07																													0
8.*M. sinensis*	0.04	0.03	0.04	0.05	0.07	0.04	0.08																												0.0018
9.*M. umbospora*	0.02	0.04	0.04	0.05	0.06	0.05	0.08	0.04																											0.0024
10.*M. zhongdongii*	0.04	0.04	0.03	0.05	0.06	0.05	0.07	0.04	0.04																										0.0038
11.*M. africana*	0.	0.	0.	0.	0.	0.	0.	0.	0.	0.																									n/c

	06	06	05	06	02	05	07	07	06	06														
	0.	0.	0.	0.	0.	0.	0.	0.	0.	0.	0.													
12.*M. alba*	03	05	02	04	06	04	07	04	03	03	06													0
	0.	0.	0.	0.	0.	0.	0.	0.	0.	0.	0.	0.												
13.*M. basicystis*	01	04	04	05	06	04	07	05	01	04	06	03												0
	0.	0.	0.	0.	0.	0.	0.	0.	0.	0.	0.	0.	0.											
14.*M. chiangmaiensis*	04	04	04	05	07	04	08	01	04	04	07	04	05											0.0055
	0.	0.	0.	0.	0.	0.	0.	0.	0.	0.	0.	0.	0.	0.										
15.*M. chumphonensis*	05	05	04	05	07	05	08	05	06	05	07	04	05	05										0.0036
	0.	0.	0.	0.	0.	0.	0.	0.	0.	0.	0.	0.	0.	0.	0.									
16.*M. disjuncta*	02	04	03	05	06	04	06	04	03	04	05	03	02	04	05									n/c
	0.	0.	0.	0.	0.	0.	0.	0.	0.	0.	0.	0.	0.	0.	0.	0.								
17.*M. epiphylla*	05	05	05	05	07	04	05	05	05	05	07	05	05	05	06	05								0.0012
	0.	0.	0.	0.	0.	0.	0.	0.	0.	0.	0.	0.	0.	0.	0.	0.	0.							
18.*M. evansii*	04	04	02	05	06	05	07	05	04	04	06	03	04	05	05	03	05							n/c
	0.	0.	0.	0.	0.	0.	0.	0.	0.	0.	0.	0.	0.	0.	0.	0.	0.	0.						
19.*M. flava*	04	03	04	05	07	04	08	02	04	04	07	04	05	02	05	04	06	05						0.0009
	0.	0.	0.	0.	0.	0.	0.	0.	0.	0.	0.	0.	0.	0.	0.	0.	0.	0.	0.					
20.*M. gracilispora*	04	03	04	05	07	04	08	01	04	05	07	04	05	02	06	04	06	05	02					0.0012
	0.	0.	0.	0.	0.	0.	0.	0.	0.	0.	0.	0.	0.	0.	0.	0.	0.	0.	0.	0.				
21.*M. insperata*	05	06	06	08	07	05	07	07	06	06	06	06	06	07	08	05	05	05	07	08				n/c
	0.	0.	0.	0.	0.	0.	0.	0.	0.	0.	0.	0.	0.	0.	0.	0.	0.	0.	0.	0.	0.			
22.*M. kanchanaburiensis*	05	04	05	05	08	04	08	01	05	04	07	04	05	01	06	04	06	05	03	01	07			0.0018
	0.	0.	0.	0.	0.	0.	0.	0.	0.	0.	0.	0.	0.	0.	0.	0.	0.	0.	0.	0.	0.	0.		
23.*M. libera*	04	04	00	04	05	04	07	04	04	03	05	02	04	04	04	03	05	02	04	04	06	05		0
24.*M. macrostroma*	0.	0.	0.	0.	0.	0.	0.	0.	0.	0.	0.	0.	0.	0.	0.	0.	0.	0.	0.	0.	0.	0.	0.	0.0276

	08	07	07	08	07	07	09	09	08	08	06	07	08	10	07	07	08	08	09	09	08	10	07												
	0.	0.	0.	0.	0.	0.	0.	0.	0.	0.	0.	0.	0.	0.	0.	0.	0.	0.	0.	0.	0.	0.	0.	0.											
25.*M. mollii*	04	04	04	04	07	04	08	00	04	04	07	04	04	01	05	04	05	05	02	01	07	01	04	09											0
	0.	0.	0.	0.	0.	0.	0.	0.	0.	0.	0.	0.	0.	0.	0.	0.	0.	0.	0.	0.	0.	0.	0.	0.	0.										
26.*M. nanensis*	04	04	03	05	07	04	08	01	04	04	07	04	04	02	05	04	05	05	02	02	07	02	03	09	01										0.0028
	0.	0.	0.	0.	0.	0.	0.	0.	0.	0.	0.	0.	0.	0.	0.	0.	0.	0.	0.	0.	0.	0.	0.	0.	0.	0.									
27.*M. nivea*	04	03	04	04	07	04	08	01	04	04	07	04	05	01	05	04	05	05	02	01	07	01	04	09	01	01									0.0009
	0.	0.	0.	0.	0.	0.	0.	0.	0.	0.	0.	0.	0.	0.	0.	0.	0.	0.	0.	0.	0.	0.	0.	0.	0.	0.	0.								
28.*M. ochracea*	04	03	04	04	07	04	08	00	04	04	07	04	04	01	05	04	05	05	02	01	07	01	04	09	00	01	01								0.0024
	0.	0.	0.	0.	0.	0.	0.	0.	0.	0.	0.	0.	0.	0.	0.	0.	0.	0.	0.	0.	0.	0.	0.	0.	0.	0.	0.	0.							
29.*M. puerensis*	04	04	00	04	06	04	07	04	04	03	05	02	04	04	04	03	05	02	04	04	06	05	00	07	04	03	04	04							0.0012
	0.	0.	0.	0.	0.	0.	0.	0.	0.	0.	0.	0.	0.	0.	0.	0.	0.	0.	0.	0.	0.	0.	0.	0.	0.	0.	0.	0.	0.						
30.*M. boliviensis*	05	05	05	05	05	04	06	06	06	06	04	05	05	06	06	05	05	05	06	06	05	06	05	05	06	06	06	06	05						n/c
	0.	0.	0.	0.	0.	0.	0.	0.	0.	0.	0.	0.	0.	0.	0.	0.	0.	0.	0.	0.	0.	0.	0.	0.	0.	0.	0.	0.	0.	0.					
31.*M. madidiensis*	04	05	04	06	06	06	08	04	05	02	06	04	05	05	05	04	06	04	05	05	07	05	04	09	04	04	04	04	04	07					0
	0.	0.	0.	0.	0.	0.	0.	0.	0.	0.	0.	0.	0.	0.	0.	0.	0.	0.	0.	0.	0.	0.	0.	0.	0.	0.	0.	0.	0.	0.	0.				
32.*M. simaoensis*	04	04	00	04	06	04	07	04	04	03	05	02	04	04	04	03	05	02	04	04	06	05	00	07	04	03	04	04	00	05	04				0.0012
	0.	0.	0.	0.	0.	0.	0.	0.	0.	0.	0.	0.	0.	0.	0.	0.	0.	0.	0.	0.	0.	0.	0.	0.	0.	0.	0.	0.	0.	0.	0.	0.			
33.*M. phukhiaoensis*	04	03	05	04	07	05	08	03	05	04	06	05	04	03	05	04	06	05	03	03	06	03	05	08	03	03	03	03	04	04	05	05			0
	0.	0.	0.	0.	0.	0.	0.	0.	0.	0.	0.	0.	0.	0.	0.	0.	0.	0.	0.	0.	0.	0.	0.	0.	0.	0.	0.	0.	0.	0.	0.	0.	0.		
34.*M. jinuoana*	05	06	06	05	05	04	07	06	05	06	06	06	05	06	07	06	05	06	06	06	06	06	05	06	06	06	06	06	05	05	08	06	06		0.0027
	0.	0.	0.	0.	0.	0.	0.	0.	0.	0.	0.	0.	0.	0.	0.	0.	0.	0.	0.	0.	0.	0.	0.	0.	0.	0.	0.	0.	0.	0.	0.	0.	0.	0.	
35.*M. longzhuensis*	05	06	05	03	07	06	08	06	06	05	07	05	05	06	06	05	06	06	06	06	07	06	05	09	06	06	06	05	05	05	07	05	06	07	0

Table S2. Interspecific genetic distance matrix and intragroup genetic distances for *tef-1α* sequences of *Moelleriella*.

Species	1	2	3	4	5	6	7	8	9	10	11	12	13	14	15	16	17	18	19	20	21	22	23	24	25	26	27	28	29	30	31	32	33	34	Genetic distance (within groups)
1.*M. phyllogena*																																			0.0202
2.*M. raciborskii*	0.13																																		0
3.*M. rhombispora*	0.11	0.12																																	0.0231
4.*M. schizostachyi*	0.11	0.12	0.11																																n/c
5.*M. sloaneae*	0.09	0.10	0.10	0.09																															0
6.*M. turbinata*	0.12	0.13	0.12	0.08	0.09																														0
7.*M.sinensis*	0.13	0.14	0.11	0.11	0.10	0.10																													0
8.*M. umbospora*	0.04	0.13	0.10	0.11	0.10	0.12	0.13																												0.0088
9.*M. zhongdongii*	0.12	0.13	0.13	0.02	0.11	0.01	0.11	0.12																											0.0202
10.*M. africana*	0.12	0.12	0.12	0.02	0.10	0.09	0.12	0.12	0.12																										n/c
11.*M. alba*	0.15	0.15	0.13	0.13	0.12	0.12	0.14	0.15	0.16	0.15																									0

	0.0	0.1	0.1	0.1	0.1	0.1	0.1	0.0	0.1	0.1	0.1													
12.*M. basicystis*	5	3	1	1	0	1	3	5	1	1	4													0.0022
13.*M.*	0.1	0.1	0.1	0.1	0.1	0.1	0.0	0.1	0.1	0.1	0.1	0.1												
chiangmaiensis	3	3	1	2	1	1	5	2	1	3	3	2												0.0011
14.*M.*	0.1	0.1	0.0	0.1	0.1	0.1	0.1	0.1	0.1	0.1	0.1	0.1	0.1											
chumphonensis	3	3	9	2	0	1	1	3	3	2	4	3	1											0
	0.0	0.1	0.1	0.1	0.0	0.1	0.1	0.0	0.1	0.1	0.1	0.0	0.1	0.1										
15.*M. disjuncta*	8	1	1	2	9	2	2	7	2	2	4	8	1	1										n/c
	0.1	0.1	0.1	0.1	0.0	0.0	0.1	0.1	0.1	0.1	0.1	0.1	0.1	0.1	0.1									
16.*M. epiphylla*	1	1	1	0	9	8	1	1	1	0	2	0	1	0	0									0.0033
	0.1	0.0	0.1	0.1	0.1	0.1	0.1	0.1	0.1	0.1	0.1	0.1	0.1	0.1	0.1	0.1								
17.*M. evansii*	3	9	3	3	1	3	5	4	4	4	5	3	4	4	3	2								n/c
	0.1	0.1	0.1	0.1	0.1	0.1	0.0	0.1	0.1	0.1	0.1	0.1	0.0	0.1	0.1	0.1	0.1							
18.*M. flava*	3	4	1	2	0	0	6	2	0	2	4	3	5	0	2	1	5							0
	0.1	0.1	0.1	0.1	0.1	0.1	0.0	0.1	0.1	0.1	0.1	0.1	0.0	0.1	0.1	0.1	0.1	0.0						
19.*M. gracilispora*	1	2	1	0	0	0	7	1	1	1	4	1	7	1	1	0	4	5						0
	0.1	0.1	0.1	0.1	0.1	0.1	0.1	0.1	0.1	0.1	0.1	0.1	0.1	0.1	0.1	0.1	0.1	0.1	0.1					
20.*M. insperata*	4	3	6	1	4	1	3	5	5	1	7	5	5	4	4	3	6	5	3					n/c
21.*M.*	0.1	0.1	0.1	0.1	0.1	0.0	0.0	0.1	0.1	0.1	0.1	0.1	0.0	0.1	0.1	0.1	0.1	0.0	0.0	0.1				
kanchanaburiensis	2	3	2	0	0	9	7	2	0	1	5	1	7	1	2	0	4	5	1	4				0
	0.1	0.0	0.1	0.1	0.1	0.1	0.1	0.1	0.1	0.1	0.1	0.1	0.1	0.1	0.1	0.1	0.0	0.1	0.1	0.1	0.1			
22.*M. libera*	3	2	2	2	0	3	4	3	3	2	5	3	3	4	1	1	9	5	2	4	3			0
	0.1	0.1	0.1	0.0	0.1	0.1	0.1	0.1	0.1	0.0	0.1	0.1	0.1	0.1	0.1	0.1	0.1	0.1	0.1	0.1	0.1	0.1		
23.*M. macrostroma*	2	3	2	9	0	0	1	1	3	9	3	2	3	1	2	1	4	2	1	3	1	3		0.0428
	0.1	0.1	0.1	0.1	0.1	0.1	0.0	0.1	0.1	0.1	0.1	0.1	0.0	0.1	0.1	0.1	0.1	0.0	0.0	0.1	0.0	0.1	0.1	
24.*M. mollii*	2	4	2	3	1	2	6	1	2	3	5	3	7	2	1	3	4	7	7	5	9	3	3	0

	0.1	0.1	0.1	0.1	0.1	0.1	0.0	0.1	0.1	0.1	0.1	0.1	0.0	0.1	0.1	0.1	0.1	0.0	0.0	0.1	0.0	0.1	0.1	0.0											
25.*M. nanensis*	4	3	2	3	1	2	5	3	1	4	4	3	5	2	2	2	5	5	7	6	7	3	3	4											0
	0.1	0.1	0.1	0.1	0.0	0.1	0.0	0.1	0.1	0.1	0.1	0.1	0.0	0.1	0.1	0.1	0.1	0.0	0.0	0.1	0.0	0.1	0.1	0.0	0.0										
26.*M. nivea*	3	3	1	1	9	1	4	3	1	3	4	3	5	0	1	2	5	5	5	4	6	3	2	4	1										0
	0.1	0.1	0.1	0.1	0.1	0.1	0.0	0.1	0.1	0.1	0.1	0.1	0.0	0.1	0.1	0.1	0.1	0.0	0.0	0.1	0.0	0.1	0.1	0.0	0.0	0.0									
27.*M. ochracea*	2	4	3	3	1	2	6	2	2	3	5	4	7	2	2	4	5	6	7	5	9	4	3	2	3	3									0.0077
	0.1	0.0	0.1	0.1	0.1	0.1	0.1	0.1	0.1	0.1	0.1	0.1	0.1	0.1	0.1	0.1	0.1	0.1	0.1	0.1	0.1	0.0	0.1	0.1	0.1	0.1	0.1								
28.*M. puerensis*	3	1	2	2	0	3	3	2	3	2	5	3	2	3	0	1	0	4	2	4	2	2	3	3	3	2	3								0.0032
	0.1	0.1	0.1	0.0	0.1	0.0	0.1	0.1	0.1	0.0	0.1	0.1	0.1	0.1	0.1	0.0	0.1	0.1	0.1	0.1	0.1	0.1	0.1	0.1	0.1	0.1	0.1	0.1							
29.*M. boliviensis*	3	3	3	8	0	9	2	3	2	9	5	3	3	4	1	9	4	3	2	4	2	2	0	4	4	4	4	2							n/c
	0.1	0.1	0.1	0.1	0.0	0.0	0.0	0.0	0.0	0.1	0.1	0.0	0.0	0.1	0.1	0.0	0.1	0.0	0.0	0.1	0.1	0.1	0.1	0.1	0.1	0.1	0.1	0.1	0.1						
30.*M. madidiensis*	0	1	0	0	8	9	8	9	7	1	2	9	9	0	1	9	1	9	9	5	0	1	0	0	0	0	0	0	0						0
	0.1	0.0	0.1	0.1	0.1	0.1	0.1	0.1	0.1	0.1	0.1	0.1	0.1	0.1	0.1	0.1	0.1	0.1	0.1	0.1	0.1	0.0	0.1	0.1	0.1	0.1	0.1	0.0	0.1	0.1					
31.*M. tanathonensis*	5	1	3	3	0	3	3	4	4	3	5	4	3	3	1	1	0	4	2	5	3	2	4	5	3	2	5	1	3	1					n/c
	0.1	0.0	0.1	0.1	0.1	0.1	0.1	0.1	0.1	0.1	0.1	0.1	0.1	0.1	0.1	0.1	0.1	0.1	0.1	0.1	0.1	0.0	0.1	0.1	0.1	0.1	0.1	0.0	0.1	0.1	0.0				
32.*M. simaoensis*	4	2	3	2	1	3	5	3	5	3	6	4	4	4	1	2	0	5	2	4	3	2	4	5	4	4	6	2	3	2	2				0
	0.1	0.1	0.1	0.1	0.1	0.1	0.1	0.1	0.1	0.1	0.1	0.1	0.1	0.1	0.1	0.1	0.1	0.1	0.1	0.1	0.1	0.1	0.1	0.1	0.1	0.1	0.1	0.1	0.1	0.1	0.1	0.1			
33.*M. jinuoana*	3	3	4	0	0	0	2	3	3	0	4	2	4	3	3	0	4	4	2	5	2	3	0	4	4	3	5	3	1	0	3	3			0.0060
	0.1	0.1	0.0	0.1	0.0	0.1	0.1	0.1	0.1	0.1	0.1	0.1	0.1	0.1	0.1	0.1	0.1	0.1	0.1	0.1	0.1	0.1	0.1	0.1	0.1	0.1	0.1	0.1	0.1	0.0	0.1	0.1	0.1		
34.*M. longzhuensis*	2	0	9	1	9	2	3	1	2	2	4	1	2	0	2	2	3	1	1	6	1	1	1	4	3	3	4	0	2	9	1	1	4		0.0059
35.*M.*	0.1	0.1	0.1	0.1	0.0	0.1	0.0	0.1	0.1	0.1	0.1	0.1	0.0	0.1	0.1	0.1	0.1	0.0	0.0	0.1	0.0	0.1	0.1	0.0	0.0	0.0	0.0	0.1	0.1	0.0	0.1	0.1	0.1	0.1	
pongdueatensis	2	1	0	1	9	0	7	1	1	1	2	2	8	0	0	0	3	7	7	3	8	1	0	9	9	8	9	1	2	8	1	2	1	1	0.0044

Table S3. Interspecific genetic distance matrix and intragroup genetic distances for *rpb1* sequences of *Moelleriella*.

Species	1	2	3	4	5	6	7	8	9	10	11	12	13	14	15	16	17	18	19	20	21	22	23	24	25	26	27	28	29	30	31	32	33	Genetic distance (within groups)
1.*M. phyllogena*																																		0.0098
	0.1																																	
2.*M. pongdueatensis*	6																																	0.0033
	0.1	0.1																																
3.*M. raciborskii*	9	8																																0.0015
	0.1	0.1	0.1																															
4.*M. rhombispora*	7	9	9																															0.0083
	0.1	0.2	0.2	0.1																														
5.*M. schizostachyi*	9	0	2	9																														n/c
	0.1	0.2	0.2	0.1	0.0																													
6.*M.sloaneae*	9	0	2	9	0																													0.0014
	0.1	0.1	0.2	0.2	0.1	0.1																												
7.*M. turbinata*	9	9	4	1	7	8																												0
	0.1	0.1	0.2	0.1	0.2	0.2	0.2																											
8.*M. sinensis*	8	1	0	9	1	1	0																											0.0057
	0.0	0.1	0.2	0.1	0.2	0.2	0.1	0.1																										
9.*M. umbospora*	2	7	0	8	0	0	9	9																										0.0014
	0.1	0.1	0.1	0.1	0.1	0.1	0.1	0.1	0.1																									
10.*M. zhongdongii*	7	3	8	9	9	9	7	4	8																									0.0055
11.*M. africana*	0.1	0.2	0.2	0.1	0.0	0.0	0.1	0.2	0.2	0.1																								n/c

	9	0	2	9	0	0	8	1	0	9														
	0.1	0.1	0.1	0.1	0.1	0.1	0.1	0.1	0.1	0.1	0.1													
12.*M. alba*	5	6	8	5	7	8	6	8	5	6	8													0
	0.0	0.1	0.1	0.1	0.1	0.1	0.1	0.1	0.0	0.1	0.1	0.1												
13.*M. basicystis*	1	6	9	7	9	9	9	9	2	7	9	5												n/c
	0.1	0.1	0.2	0.1	0.2	0.2	0.2	0.0	0.1	0.1	0.2	0.1	0.1											
14.*M. chiangmaiensis*	8	0	0	9	2	2	0	4	9	5	2	7	7											0
	0.1	0.1	0.1	0.1	0.1	0.1	0.2	0.1	0.1	0.1	0.1	0.1	0.1	0.1										
15.*M. chumphonensis*	4	7	9	5	9	9	0	8	5	7	9	6	4	7										0.0071
	0.2	0.2	0.2	0.2	0.1	0.1	0.1	0.2	0.2	0.2	0.1	0.1	0.2	0.2	0.1									
16.*M. epiphylla*	1	3	4	2	6	6	3	5	1	0	7	9	0	2	9									0.0031
	0.1	0.1	0.1	0.1	0.1	0.1	0.1	0.1	0.1	0.1	0.1	0.1	0.1	0.1	0.0	0.1								
17.*M. evansii*	4	7	8	5	8	8	9	8	5	7	8	5	4	7	0	9								n/c
	0.1	0.1	0.2	0.2	0.1	0.1	0.1	0.0	0.1	0.1	0.1	0.1	0.1	0.0	0.1	0.2	0.1							
18.*M. flava*	6	1	0	0	9	9	8	7	7	4	9	7	7	6	6	1	6							0
	0.1	0.1	0.2	0.1	0.1	0.1	0.2	0.0	0.1	0.1	0.1	0.1	0.1	0.0	0.1	0.2	0.1	0.0						
19.*M. gracilispora*	8	3	1	8	9	9	0	9	9	4	9	7	8	9	8	2	8	7						0
	0.2	0.2	0.2	0.2	0.2	0.2	0.1	0.2	0.2	0.2	0.2	0.1	0.2	0.2	0.2	0.1	0.2	0.2	0.2					
20.*M. insperata*	2	3	4	2	0	0	5	4	3	0	0	9	2	5	2	7	1	1	3					n/c
	0.1	0.1	0.2	0.1	0.1	0.1	0.2	0.0	0.1	0.1	0.1	0.1	0.1	0.0	0.1	0.2	0.1	0.0	0.0	0.2				
21.*M. kanchanaburiensis*	7	2	2	9	9	9	0	9	8	5	9	7	8	9	6	1	6	8	2	1				0
	0.2	0.2	0.2	0.2	0.1	0.1	0.1	0.2	0.2	0.2	0.1	0.1	0.2	0.2	0.2	0.1	0.2	0.2	0.2	0.1	0.2			
22.*M. libera*	1	6	4	3	7	7	4	5	1	2	7	8	1	3	2	1	1	4	3	8	3			0
	0.2	0.2	0.2	0.2	0.1	0.1	0.1	0.2	0.2	0.2	0.1	0.1	0.2	0.2	0.2	0.1	0.1	0.2	0.2	0.1	0.2	0.1		
23.*M. macrostroma*	1	3	2	0	8	7	5	2	2	0	7	7	1	1	0	5	9	0	2	3	1	6		0.0168
24.*M. mollii*	0.1	0.1	0.1	0.1	0.2	0.2	0.2	0.0	0.1	0.1	0.2	0.1	0.1	0.0	0.1	0.2	0.1	0.0	0.0	0.2	0.0	0.2	0.2	0

	8	3	9	9	0	0	2	4	9	3	0	7	9	4	8	4	8	6	8	4	9	4	3											
	0.1	0.1	0.2	0.2	0.2	0.2	0.2	0.0	0.1	0.1	0.2	0.1	0.1	0.0	0.1	0.2	0.1	0.0	0.0	0.2	0.1	0.2	0.2	0.0										
25.*M. nanensis*	8	2	0	0	1	1	1	3	9	4	1	8	9	4	9	5	9	6	9	5	0	5	2	1										0
	0.1	0.1	0.2	0.2	0.2	0.2	0.2	0.0	0.1	0.1	0.2	0.1	0.1	0.0	0.1	0.2	0.1	0.0	0.0	0.2	0.1	0.2	0.2	0.0	0.0									
26.*M. nivea*	8	2	0	0	1	1	1	3	9	4	1	8	9	5	9	4	9	7	9	4	0	5	2	2	1									0.0011
	0.1	0.1	0.1	0.1	0.2	0.2	0.2	0.0	0.1	0.1	0.2	0.1	0.1	0.0	0.1	0.2	0.1	0.0	0.0	0.2	0.0	0.2	0.2	0.0	0.0	0.0								
27.*M. ochracea*	8	2	9	9	0	0	1	3	9	2	0	7	9	4	8	5	7	7	9	4	9	5	2	1	1	2								0.0069
	0.1	0.1	0.0	0.2	0.2	0.2	0.2	0.2	0.2	0.1	0.2	0.2	0.1	0.2	0.1	0.2	0.1	0.2	0.2	0.2	0.2	0.2	0.2	0.2	0.2	0.2	0.2							
28.*M. puerensis*	9	8	1	1	3	3	5	0	0	8	3	0	9	0	9	5	9	1	1	5	2	5	4	0	0	1	1							0
	0.2	0.2	0.2	0.2	0.2	0.2	0.1	0.2	0.2	0.2	0.2	0.1	0.2	0.2	0.2	0.1	0.2	0.2	0.2	0.0	0.2	0.1	0.1	0.2	0.2	0.2	0.2	0.2						
29.*M. boliviensis*	2	3	4	4	1	0	5	4	2	1	0	9	2	4	0	6	0	1	3	2	1	7	3	6	5	4	6	4						n/c
	0.1	0.1	0.1	0.1	0.1	0.1	0.1	0.1	0.1	0.0	0.1	0.1	0.1	0.1	0.1	0.2	0.1	0.1	0.1	0.2	0.1	0.2	0.1	0.1	0.1	0.1	0.1	0.1	0.2					
30.*M. madidiensis*	6	2	8	8	9	9	5	3	7	7	9	3	7	4	7	0	7	3	4	0	5	2	8	3	4	4	2	9	0					0
	0.2	0.1	0.0	0.2	0.2	0.2	0.2	0.2	0.2	0.2	0.2	0.2	0.2	0.2	0.2	0.2	0.2	0.2	0.2	0.2	0.2	0.2	0.2	0.2	0.2	0.2	0.2	0.0	0.2	0.2				
31.*M. simaoensis*	1	9	2	2	4	4	6	3	2	0	4	1	2	2	1	7	1	3	3	6	3	7	5	3	2	3	3	2	4	0				0
	0.1	0.1	0.2	0.1	0.2	0.2	0.1	0.1	0.2	0.1	0.2	0.1	0.1	0.1	0.2	0.2	0.2	0.0	0.1	0.2	0.1	0.2	0.2	0.1	0.1	0.1	0.1	0.2	0.2	0.1	0.2			
32.*M. phukhiaoensis*	9	1	2	9	0	0	8	1	0	4	1	6	9	0	0	4	0	8	3	2	1	3	1	2	1	1	2	2	1	4	3			0
	0.2	0.2	0.2	0.2	0.1	0.1	0.1	0.2	0.2	0.2	0.1	0.1	0.2	0.2	0.1	0.1	0.1	0.2	0.2	0.1	0.2	0.1	0.1	0.2	0.2	0.2	0.2	0.2	0.1	0.1	0.2	0.2		
33.*M. jinuoana*	0	2	4	2	5	5	5	3	2	0	5	7	0	2	8	5	8	0	0	5	1	5	5	2	2	2	2	5	5	9	6	0		0.0038
	0.1	0.1	0.1	0.0	0.2	0.2	0.2	0.1	0.1	0.1	0.2	0.1	0.1	0.1	0.1	0.2	0.1	0.1	0.1	0.2	0.1	0.2	0.2	0.1	0.1	0.1	0.1	0.1	0.2	0.1	0.2	0.1	0.2	
34.*M. longzhuensis*	7	9	9	8	0	0	2	8	7	7	0	6	7	8	3	1	3	8	9	4	8	3	0	8	8	8	8	9	3	7	0	8	0	0.0033

Table S4. Interspecific genetic distance matrix and intragroup genetic distances for nr*LSU* sequences of *Conoideocrella*.

Species	1	2	3	Genetic distance (within groups)
1.*Conoideocrella luteorostrata*				0.0012
2.*C. tenuis*	0.02			0.0028
3.*C. fenshuilingensis*	0.02	0.01		0
4.*C. krungchingensis*	0.03	0.02	0.02	0.0007

Table S5. Interspecific genetic distance matrix and intragroup genetic distances for *tef-1α* sequences of *Conoideocrella*.

Species	1	2	3	Genetic distance (within groups)
1.*C. luteorostrata*				n/c
2.*C. tenuis*	0.03			0.0052
3.*C. fenshuilungensis*	0.06	0.06		0
4.*C. krungchingensis*	0.08	0.07	0.09	0.0011

Table S6. Interspecific genetic distance matrix and intragroup genetic distances for *rpb1* sequences of *Conoideocrella*.

Species	1	2	Genetic distance (within groups)
1.*C. luteorostrata*			0.0019
2.*C. tenuis*	0.05		0.0023
3.*C. fenshuilingensis*	0.08	0.08	0.0027
